# Supplementary material for: Mapping the evolution of fertility support policies in China: A content and instrumental analysis
Source: PLoS One. 2025 Oct 9;20(10):e0332137. doi: 10.1371/journal.pone.0332137 (PMC12510515; doi:10.1371/journal.pone.0332137)
Supplement: S1 Appendix — (ZIP) [file pone.0332137.s001.zip › S1 Appendix. 226 original policy documents/29-国家卫生健康委办公厅关于开展分娩镇痛试点工作的通知(FBM-CLI-4-326465).doc]

国家卫生健康委办公厅关于开展分娩镇痛试点工作的通知

**发布部门：** [国家卫生健康委员会](https://www.pkulaw.com/law/chl?Aggs.IssueDepartment=60321) [机构沿革](javascript:void(0))

**发文字号：**国卫办医函〔2018〕1009号

**发布日期：**2018.11.15

**实施日期：**2018.11.15

**时效性：** [现行有效](https://www.pkulaw.com/law/chl?Aggs.TimelinessDic=01)

**效力级别：** [部门工作文件](https://www.pkulaw.com/law/chl?Aggs.EffectivenessDic=XE0303)

**法规类别：** [卫生综合规定](https://www.pkulaw.com/law/chl?Aggs.Category=09601)

国家卫生健康委办公厅关于开展分娩镇痛试点工作的通知
（国卫办医函〔2018〕1009号）

各省、自治区、直辖市及新疆生产建设兵团卫生健康委（卫生计生委）：
　　为贯彻落实《关于印发[加强和完善麻醉医疗服务意见的通知](https://www.pkulaw.com/chl/78ca6b86c27f73a7bdfb.html)》（国卫医发〔2018〕21号）精神，进一步规范分娩镇痛相关诊疗行为，提升产妇分娩镇痛水平，提高围产期医疗服务质量，我委决定在全国开展分娩镇痛试点工作。现将工作方案印发给你们，请遵照执行。各地卫生健康行政部门要切实加强组织领导，结合实际认真组织实施，确保试点工作顺利开展，并及时将试点有关情况报送我委。
　　国家卫生健康委联系人：医政医管局 丁雯、王斐
　　联系电话：010-68791885、68791889
　　中国医师协会联系人：全科与继教部 王振华
　　联系电话：010-63318368

国家卫生健康委办公厅
2018年11月15日

　　分娩镇痛试点工作方案（2018-2020年）

　　为进一步规范分娩镇痛相关诊疗行为，提升产妇分娩诊疗水平，优化与完善医院分娩镇痛的整体管理流程，提高围产期医疗服务质量，制定本方案。
　　**一、**工作目标
　　2018-2020年，在全国范围内遴选一定数量的医院开展分娩镇痛诊疗试点工作（以下简称试点医院）。发挥试点医院的带动和示范作用，以点带面，逐步在全国推广分娩镇痛的诊疗工作。进一步规范分娩镇痛操作技术，提升分娩镇痛的覆盖范围，普及镇痛条件下的自然分娩，降低剖宫产率，增强医疗服务舒适化程度，提高孕产妇就医满意度。加强产房医师团队的急救能力，进一步保障孕产妇安全，降低孕产妇死亡，进一步增强人民群众看病就医获得感、幸福感。

　　**二、**试点范围
　　具备产科和麻醉科诊疗科目的二级及以上综合医院、妇幼保健院或妇产专科医院。

　　**三、**组织管理
　　我委医政医管局负责制定试点工作方案，提出试点医院认定和考核条件，并指导各地开展试点工作。
　　各省级卫生健康行政部门负责组织实施本地分娩镇痛试点，开展试点医院遴选、认定、培训、指导以及考核等工作。
　　受我委委托，中国医师协会麻醉学医师分会、妇产科医师分会会同中华医学会麻醉学分会，成立分娩镇痛试点专家工作组（以下简称专家工作组），协助我委制订分娩镇痛技术操作规范和管理规范，为地方卫生健康行政部门开展试点工作提供技术支持。

　　**四、**试点内容和要求
　　试点医院应当进一步规范分娩镇痛技术操作，不断完善优化分娩镇痛管理和服务流程，提高医务人员及公众对分娩镇痛的认知度，在保障母婴安全的前提下，普及分娩镇痛技术。重点做好以下工作：
　　（一）提高分娩镇痛技术水平。
　　1.按照《分娩镇痛技术操作规范》（见附件1）及《分娩镇痛技术管理规范》（见附件2）的要求，建立分娩镇痛的相关管理制度，完善工作机制，优化服务流程，提高分娩镇痛医疗质量和安全。
　　2.开展医护人员定期培训，提高医护人员分娩镇痛的理论水平和操作能力。
　　（二）提高产妇分娩方式选择的科学性。
　　通过实施分娩镇痛，降低产妇因不能耐受分娩疼痛而行剖宫产分娩的比例，推动剖宫产率逐步下降，提高自然分娩率，进一步提升产妇和新生儿健康水平。
　　（三）增强医院和相关科室对分娩镇痛的认识。
　　1.医院应当为分娩镇痛工作提供必要的支持，包括人员、设备设施和必要的政策支持，调动医务人员开展分娩镇痛工作的积极性。
　　2.医院应当建立分娩镇痛管理服务团队，加强对相关专业医务人员（麻醉科医师、产科医师、儿科医师、助产士、麻醉科护士、产科护士）的培训和临床演练及考核，使其熟练掌握各种相关紧急情况的应对预案，以及“即刻剖宫产/急救复苏”等紧急情况的处理流程。注意并发症监测和防治能力建设，应当有针对性地建立健全分娩镇痛质量控制体系，提高分娩镇痛的安全性。
　　3.相关科室负责人应当加强对分娩镇痛工作的管理和监督，指定专人定期对分娩镇痛实施情况和存在的问题进行评估和反馈，不断改进工作方法，保证分娩镇痛的安全性，提高产妇的舒适度和满意度。
　　（四）加强对孕产妇以及家属的健康宣教。
　　建立孕产妇及其家属的定期宣教制度，采用孕妇学校授课、网络宣传推广、发放宣教手册、设立宣教栏等多种形式、撰写科普书籍，在报纸、广播、电视、互联网等各类媒体进行科普宣传，提高产妇和家属对产痛危害的认识，提高对分娩镇痛的认知度。
　　（五）发挥试点医院示范带动作用。
　　1.专家工作组组织“分娩镇痛全国专家巡讲团”，协助地方卫生健康行政部门对试点医院相关科室医务人员进行培训，并组织临床示教。
　　2.各试点医院应当定期组织分娩镇痛规范化培训，大力推广分娩镇痛理念，推进试点医院规范开展分娩镇痛。同时，通过帮扶、协作、接收进修等形式，将分娩镇痛技术向其医联体内其他医疗机构推广，发挥试点医院辐射带动作用。

　　**五、**实施步骤
　　（一）筹备启动阶段（2018年11-12月）
　　1.我委制定印发试点工作方案。
　　2.省级卫生健康行政部门根据试点方案及试点医院标准（见附件3），组织审核认定试点医院，并将试点医院名单报送我委医政医管局。
　　（二）组织实施阶段（2019年1月-2020年9月）
　　1.我委印发试点医院名单，各试点医院按照试点工作方案要求开展工作。
　　2.省级卫生健康行政部门定期组织对辖区内试点医院进行培训、指导、评估和考核工作（考核要求见附件3）。专家工作组做好相应技术支持工作。
　　（三）总结评估阶段（2020年9月-2020年12月）
　　省级卫生健康行政部门组织对本辖区试点工作进行自评估；我委组织对全国试点工作进行总结评估，宣传和推广先进典型和经验，进一步在全国推广分娩镇痛。

　　**六、**工作要求
　　（一）提高思想认识、加强组织领导。各级卫生健康行政部门要统一思想，从推动医疗服务高质量发展和不断满足人民日益增长的美好生活需要的高度，充分认识开展分娩镇痛服务的重要性，切实加强组织领导，并积极协调相关部门为试点工作提供收费、医保报销等政策支持。
　　（二）认真贯彻落实、扎实稳步推进。各级卫生健康行政部门和试点医院要认真贯彻落实文件精神，明确职责，结合本地实际认真组织实施。鼓励试点医院结合自身实际，在确保安全的前提下，创造性地开展工作，探索建立分娩镇痛服务的长效机制。
　　（三）加强监督指导、确保取得实效。地方各级卫生健康行政部门要适时开展检查指导，认真组织试点评估工作。我委将适时组织对试点工作进行评估，确保试点工作落到实处。
　　附件：1.分娩镇痛技术操作规范
　　2.分娩镇痛技术管理规范
　　3.试点医院基本条件与考核要求
　　4.分娩镇痛试点专家工作组成员名单
　　附件1：

分娩镇痛技术操作规范

　　为规范分娩镇痛技术的临床应用，保证医疗质量与医疗安全，制定本规范，作为医疗机构及其医师开展分娩镇痛诊疗技术的操作要求。本规范所称的分娩镇痛技术，是指椎管内分娩镇痛技术，主要包括硬膜外腔镇痛、蛛网膜下腔镇痛和蛛网膜下腔-硬膜外腔联合镇痛等诊疗技术。

　　**一、**椎管内分娩镇痛实施前产妇的评估
　　椎管内分娩镇痛前对产妇系统的评估是保证镇痛安全及顺利实施的基础。评估内容主要包括病史（现病史、既往史、麻醉手术史、药物过敏史、合并症、特殊药物应用等）、体格检查，以及相关实验室检查（包括血常规和血小板计数、凝血功能检查等）。存在合并症或其他异常情况者，应当进行相应的特殊实验室检查。

　　**二、**椎管内分娩镇痛适应证和禁忌证
　　（一）椎管内分娩镇痛的适应证。
　　1.产妇自愿应用。
　　2.经产科医师评估，可阴道分娩或经阴道试产者。
　　（二）椎管内分娩镇痛的禁忌证。
　　1.产妇不同意，拒绝签署知情同意者。
　　2.存在椎管内阻滞禁忌证者，如凝血功能异常、穿刺部位感染或损伤、低血容量或低血压、颅内压增高、脊柱病变或严重脊柱畸形，神经系统疾病或神经病变等。
　　3.对局部麻醉药及阿片类药物过敏者。
　　4.产妇无法配合进行穿刺的情况。

　　**三、**椎管内分娩镇痛的知情同意
　　椎管内分娩镇痛的实施应该由产妇本人自愿同意，由产妇本人或其委托代理人签署知情同意书后方能实施。椎管内分娩镇痛知情同意书应当详细描述其预期的效果、优势，对产程和胎儿的可能影响，潜在的并发症及不良反应，以及可能的替代手段，由实施操作的麻醉医师向产妇进行详细说明后签署。

　　**四、**椎管内分娩镇痛开始的时机
　　产程开始后，产妇有要求，经评估无禁忌证后，在产程的任何阶段均可开始实施椎管内分娩镇痛。

　　**五、**椎管内分娩镇痛实施流程
　　椎管内分娩镇痛的实施可参考下列流程：
　　1.产程开始后，产妇提出要求。
　　2.产科医师/助产士/产房护士、麻醉科医师进行评估。
　　3.拟定镇痛方式。
　　4.签署知情同意。
　　5.准备相关物品，建立生命体征监测及胎心监测。
　　6.开放静脉通路。
　　7.实施椎管内镇痛操作。
　　8.对椎管内分娩镇痛进行管理。
　　9.分娩镇痛结束，观察2小时后返病房。
　　10.随访，注意观察镇痛后恢复情况，包括下肢感觉、运动恢复情况及排尿情况等。

　　**六、**椎管内分娩镇痛实施方法
　　（一）硬膜外腔镇痛。
　　1.穿刺前需要开放静脉通路及建立产妇的生命体征监测。
　　2.选择腰2-3或腰3-4间隙，严格按椎管内穿刺操作流程进行硬膜外腔穿刺，向头端置入硬膜外导管，确保3-5cm进入硬膜外腔。
　　3.连接无菌注射器轻柔回抽确认无血液和脑脊液流出后，经硬膜外导管注入试验剂量1-1.5%利多卡因3-5ml（总量≤50mg），观察3-5分钟，排除导管置入血管或蛛网膜下腔的可能；注入首剂量6-15ml低浓度局部麻醉药和阿片类药物混合液。
　　4.将导管牢固地固定于产妇背部，与患者自控镇痛泵系统相连，进行连续给药；应用尖端柔软的钢丝加强硬膜外导管可以降低硬膜外导管置入血管的可能。
　　5.硬膜外腔分娩镇痛药物宜联合应用低浓度局部麻醉药和阿片类药物。推荐药物为：低浓度局麻药（如0.04-0.125%布比卡因或0.0625-0.15%罗哌卡因）联合应用脂溶性阿片类药物（如芬太尼1-2μg/ml或舒芬太尼0.4-0.6μg/ml）。
　　6.镇痛维持阶段建议使用患者自控镇痛泵，患者自控给药联合持续背景输注或程序化脉冲式硬膜外腔给药是较好的选择。根据疼痛程度调整镇痛泵的设置（4-10ml/h）。
　　7.首次给予负荷剂量后应测量镇痛平面（理想状况应维持在胸10水平），进行视觉模拟疼痛评分和Bromage运动神经阻滞评估。
　　8.由麻醉医生完成相应操作记录，协同产科医生及助产士/产房护士观察并处理分娩镇痛过程中的情况。
　　（二）蛛网膜下腔镇痛。
　　1.准备同硬膜外腔分娩镇痛。
　　2.选择腰3-4（首选）或腰2-3间隙进行蛛网膜下腔穿刺。
　　3.经腰麻针注入镇痛药，如罗哌卡因2-4mg或布比卡因1.25-2.5mg联合芬太尼10-15μg或舒芬太尼2.5-5μg；退出腰麻针（及硬膜外穿刺针）。
　　4.单次蛛网膜下腔阻滞镇痛可维持镇痛效果1-2小时。连续蛛网膜下腔镇痛不做推荐常规用于分娩镇痛。
　　（三）蛛网膜下腔-硬膜外腔联合镇痛。
　　1.准备同硬膜外腔分娩镇痛。
　　2.选择腰3-4（首选）或腰2-3间隙进行硬膜外腔穿刺和蛛网膜下腔穿刺。
　　3.经腰麻针注入镇痛药，如罗哌卡因2-4mg或布比卡因1.25-2.5mg联合芬太尼10-15μg或舒芬太尼2.5-5μg；退出腰麻针后，向头侧置入硬膜外导管。
　　4.硬膜外腔给药前回抽，确认无血及脑脊液。经硬膜外导管注入试验剂量1-1.5%利多卡因3-5ml（总量≤50mg），观察3-5分钟，排除硬膜外导管置入血管或蛛网膜下腔可能。
　　5.镇痛管理同硬膜外腔镇痛。

　　**七、**椎管内分娩镇痛期间的镇痛和产程管理
　　（一）椎管内分娩镇痛开始后，麻醉医师应根据产妇镇痛效果及其他相关情况随时调整处理，理想状况下宜将分娩期疼痛控制在视觉模拟评分≤3分。
　　（二）分娩镇痛期间应当进行生命体征监测和胎心监测：试验剂量注入后麻醉医生应每5-10分钟监测一次直至首剂量注入后半小时；如处理分娩镇痛爆发痛后给予追加剂量，应每5-10分钟监测一次直至半小时。
　　（三）助产士/产房护士应对产程进展、胎心情况及产妇生命体征常规监测及管理。
　　（四）分娩镇痛中爆发痛的处理：应对产妇疼痛的性质和部位进行评估，评估硬膜外导管的位置和给药设备的功能状况，检查阻滞的感觉平面。根据产程进展及产妇需求，决定是否需要调整硬膜外腔药物剂量和浓度或给予硬膜外腔追加剂量；追加剂量应避免使用高浓度局麻药物，以免产生运动神经阻滞；必要时重新穿刺更换硬膜外导管。
　　（五）椎管内分娩镇痛宜实施全产程镇痛。分娩结束后观察2小时，产妇无异常情况离开产房时，拔除硬膜外导管返回病房。

　　**八、**椎管内分娩镇痛并发症的处理
　　（一）低血压。评估低血压产生原因，除外产科因素。同时嘱产妇左侧卧位，给予吸氧、输液，根据情况给予适量麻黄碱或去氧肾上腺素。
　　（二）胎心率异常。评估原因，嘱产妇左侧卧位，给予吸氧、连续胎心监测，排除及处理母体低血压因素；暂停缩宫素；持续观察胎心变异情况，必要时进行紧急剖宫产。
　　（三）镇痛不全或偏侧阻滞。排除产科因素；判断硬膜外导管是否在位及是否打折受压；调整硬膜外导管位置；硬膜外腔给予补充剂量的局部麻醉药物；必要时重新进行硬膜外腔穿刺。
　　（四）瘙痒。蛛网膜下腔给予阿片类药物镇痛的产妇相对多见。根据具体情况，可给予小剂量阿片受体拮抗剂进行治疗，如纳洛酮40-80μg等。
　　（五）恶心呕吐。低血压引起的恶心呕吐应首先治疗低血压；还可使用5-羟色胺3受体拮抗剂进行治疗。
　　（六）尿潴留。助产士/产房护士应动态监测产妇膀胱充盈情况，必要时实施导尿。
　　（七）发热。分娩镇痛期间出现发热的产妇应进行常规退热处理，适量进行补液。应除外感染因素，根据感染情况决定抗生素的使用及是否需要立即终止妊娠。
　　（八）呼吸抑制。罕见，常由阿片类药物引起，可给予纳洛酮进行拮抗。
　　（九）局部麻醉药全身中毒反应。如果产妇出现了局部麻醉药中枢神经系统中毒的症状和体征，应立即停止注入局部麻醉药物，使用较低剂量的苯二氮卓类药物治疗抽搐发作；同时给氧，对产妇进行保护。如出现局部麻醉药心血管毒性反应，应启动复苏流程；静脉用20%脂肪乳剂是局部麻醉药心血管毒性反应的有效解毒剂，可及时使用。根据产妇和胎儿情况，必要时考虑行紧急剖宫产终止妊娠，并开始新生儿复苏。
　　（十）高位脊髓阻滞。见于拟注入硬膜外腔的药物误入蛛网膜下腔，严重者可致心跳骤停。采用标准复苏程序予以复苏。

　　**九、**椎管内分娩镇痛期间的液体管理
　　（一）在分娩镇痛开始之前应开放静脉通路，并维持至分娩结束。
　　（二）分娩期间液体管理：椎管内分娩镇痛期间应维持液体输注；根据产妇是否严格禁食水，是否合并下列情况（如低血压、发热、恶心呕吐等）或疾病（如妊娠高血压综合征、妊娠期糖尿病、心脏或肾脏疾患等），决定是否给予含糖液输注及液体输注的速度；注意监控产妇尿量，避免液体过负荷。

　　**十、**椎管内分娩镇痛期间的饮食管理
　　产妇在进入产房接受分娩镇痛后宜避免摄入高油、高蛋白质的固体食物，推荐饮用高能量无渣饮料。

　　**十一、**椎管内分娩镇痛产妇由自然分娩转为剖宫产的处理原则
　　根据剖宫产的紧急程度及硬膜外腔分娩镇痛的效果选择紧急剖宫产的麻醉方式。可选择从现有硬膜外导管给予碳酸利多卡因、利多卡因或氯普鲁卡因进行硬膜外腔麻醉；或者重新穿刺进行蛛网膜下腔麻醉；或选择全身麻醉。

　　**十二、**椎管内分娩镇痛操作记录
　　椎管内分娩镇痛操作完成后应进行详细记录。记录内容应涵盖评估、操作、镇痛效果和安全性评价、并发症及随访。记录内容应持续至分娩镇痛结束。在椎管内分娩镇痛给予负荷剂量的30分钟内，应每5-10分钟记录产妇的生命体征，之后应每0.5-2小时视产妇情况记录生命体征。胎心监护应至少持续至首次剂量给予后20分钟。此记录应作为病历的一部分进行保存。
　　附件2：

分娩镇痛技术管理规范

　　为规范分娩镇痛技术的临床应用，保证医疗质量与医疗安全，制定本规范，作为医疗机构及其医师开展椎管内分娩镇痛诊疗技术的基本要求。本规范所称的分娩镇痛技术，是指椎管内分娩镇痛技术，主要包括硬膜外腔镇痛、蛛网膜下腔镇痛、蛛网膜下腔-硬膜外腔联合镇痛等诊疗技术。

　　**一、**医疗机构基本要求
　　（一）医疗机构开展椎管内分娩镇痛应当与其功能、任务相适应。
　　（二）二级、三级综合医院、妇幼保健院或者妇产专科医院，有卫生健康行政部门核准登记的与椎管内分娩镇痛相关的诊疗科目。
　　（三）对开展椎管内分娩镇痛的医疗机构设备、设施的基本要求：
　　1.具备实施椎管内分娩镇痛临床工作的基本设施：具有完善消毒条件的独立操作空间，按照院内感染控制制度对产房的要求进行院感监测与管理。
　　2.具备实施椎管内分娩镇痛临床工作的基本设备：多功能监护仪；供氧设备包括中心供氧/氧气瓶、鼻吸氧管、吸氧面罩；吸引设备包括负压吸引器、吸引管、吸痰管；椎管内镇痛穿刺包、镇痛泵；胎心监护仪、新生儿抢救复苏设备；抢救车，包括抢救物品及药品；气管插管设备包括喉镜、气管导管、口咽通气管、喉罩、困难气道器具等；医疗区域内具有麻醉机和除颤器等。抢救设备由专人负责维护、定期检查并做登记。
　　3.具有实施椎管内分娩镇痛的基本药品和处理意外或并发症的应急药品：静脉输液用液体；局部麻醉药（利多卡因、罗哌卡因、布比卡因等）；阿片类药物（芬太尼、舒芬太尼等）；急救类药品（麻黄素、阿托品、去氧肾上腺素、肾上腺素、咪达唑仑、脂肪乳剂等）；消毒液。毒麻药管理按照国家规范要求执行。所有药品由专人负责维护补充、定期检查并做登记。

　　**二、**人员基本要求
　　（一）麻醉科医师。
　　1.取得医师执业证书，执业范围为麻醉科专业，执业地点为申请单位。
　　2.3年以上高年住院医师及以上职称，经科室评估具备独立从事分娩镇痛的能力。
　　3.具有毒麻类药品处方权。
　　（二）其他卫生专业技术人员。配合实施椎管内分娩镇痛的相关护理人员、产科医师应当取得护士执业证书、产科医师资格证书、执业证书，并经椎管内分娩镇痛相关系统培训。

　　**三、**技术管理基本要求
　　（一）严格遵守椎管内分娩镇痛技术操作规范，掌握椎管内分娩镇痛的适应证和禁忌证。
　　（二）实施椎管内分娩镇痛必须使用经国家药品监督管理部门注册的专业设备、耗材和药品。
　　（三）椎管内分娩镇痛应由具有椎管内分娩镇痛临床实施能力的麻醉专业医师实施。实施前应当全面评估产妇病情，制定完善的镇痛方案，并具备预防并发症的措施。
　　（四）实施椎管内分娩镇痛前，必须向患者或其法定监护人、代理人告知椎管内分娩镇痛技术的风险、获益、围镇痛期注意事项、可能发生的并发症和预防措施等，并签署知情同意书。
　　（五）加强椎管内分娩镇痛的质量管理，建立健全的镇痛后随访制度，并按规定进行随访、记录。
　　（六）医疗机构和医师应当按规定接受卫生健康行政部门或相关专业质控中心的检查评估，包括患者选择、并发症发生情况、医疗事故发生情况、随访情况等。
　　（七）临床医师实施椎管内分娩镇痛需按规定及时填写、签署医学文书，不得隐匿、伪造、销毁医学文书和有关资料。
　　（八）其他管理要求。
　　1.建立椎管内分娩镇痛药品、耗材登记制度，保证相关药品、耗材来源可追溯。
　　2.不得重复使用一次性耗材。
　　3.严格执行国家物价、财务政策制度。
　　附件3：

试点医院基本条件与考核要求

　　**一、**基本条件
　　（一）二级及以上综合医院或妇产医院。
　　（二）具备产科、麻醉科诊疗科目。
　　（三）具备开展分娩镇痛的专业技术人员。
　　（四）具有良好的椎管内分娩镇痛基础，椎管内分娩镇痛率≥10%。
　　（五）医院给予必要的人员、硬件和政策支持。

　　**二、**考核要求
　　试点医院应当在2020年底前达到以下要求：
　　（一）分娩镇痛技术操作和管理符合规范要求。
　　（二）椎管内分娩镇痛率≥40%。
　　附件4：

分娩镇痛试点专家工作组成员名单
（按姓氏笔画排序）

　　**一、**组长
　　米卫东　　中国医师协会麻醉学医师分会会长、中国人民解放军总医院主任医师
　　郎景和　　中国医师协会妇产科医师分会会长、中国工程院院士、北京协和医院主任医师
　　黄宇光　　中华医学会麻醉学分会主任委员、北京协和医院主任医师

　　**二、**副组长
　　张为远　　首都医科大学附属北京妇产医院　　　　　主任医师
　　陈敦金　　广州医科大学附属第三医院　　　　　　　主任医师
　　姚尚龙　　华中科技大学同济医学院附属协和医院　　主任医师

　　**三、**成员
　　丁依玲　　中南大学湘雅二医院　　　　　　　　　　主任医师
　　万小平　　上海第一妇婴保健院　　　　　　　　　　主任医师
　　马玉燕　　山东大学齐鲁医院　　　　　　　　　　　主任医师
　　王　丹　　陆军军医大学第一附属医院　　　　　　　主任医师
　　王东信　　北京大学第一医院　　　　　　　　　　　主任医师
　　王世宣　　华中科技大学同济医学院附属同济医院　　主任医师
　　古　航　　海军军医大学长海医院　　　　　　　　　主任医师
　　吕改华　　山西省儿童医院　　　　　　　　　　　　主任医师
　　刘　薇　　北京和睦家医院　　　　　　　　　　　　主任医师
　　刘志强　　上海市第一妇婴保健院　　　　　　　　　主任医师
　　刘心国　　济南市第五人民医院　　　　　　　　　　主任医师
　　刘彩霞　　中国医科大学附属盛京医院　　　　　　　主任医师
　　李佩玲　　哈尔滨医科大学附属第二医院　　　　　　主任医师
　　李爱媛　　湖南省妇幼保健院　　　　　　　　　　　主任医师
　　宋兴荣　　广州市妇女儿童医疗中心　　　　　　　　主任医师
　　连庆泉　　温州医科大学附属第二医院　　　　　　　主任医师
　　张小兰　　甘肃省妇幼保健院　　　　　　　　　　　主任医师
　　张　瑾　　石家庄市第四医院　　　　　　　　　　　主任医师
　　杨　孜　　北京大学第三医院　　　　　　　　　　　主任医师
　　杨慧霞　　北京大学第一医院　　　　　　　　　　　主任医师
　　沈晓凤　　南京医科大学附属南京妇产医院　　　　　主任医师
　　陈　叙　　天津中心妇产科医院　　　　　　　　　　主任医师
　　张析哲　　赤峰市医院　　　　　　　　　　　　　　主任医师
　　林　元　　福建省妇幼保健院　　　　　　　　　　　主任医师
　　林　俊　　浙江大学医学院附属妇产科医院　　　　　主任医师
　　林雪梅　　四川大学华西第二医院　　　　　　　　　主任医师
　　赵　平　　中国医科大学附属盛京医院　　　　　　　主任医师
　　胡祖荣　　广东省妇幼保健院　　　　　　　　　　　主任医师
　　胡娅莉　　南京鼓楼医院　　　　　　　　　　　　　主任医师
　　钟　梅　　南方医科大学南方医院　　　　　　　　　主任医师
　　郭瑞霞　　郑州医科大学第一附属医院　　　　　　　主任医师
　　徐铭军　　首都医科大学附属北京妇产医院　　　　　主任医师
　　徐丛剑　　复旦大学附属妇产科医院　　　　　　　　主任医师
　　高和新　　新疆维吾尔自治区妇幼保健院　　　　　　主任医师
　　黄绍强　　复旦大学附属妇产科医院　　　　　　　　主任医师
　　黄向华　　河北医科大学第二医院　　　　　　　　　主任医师
　　龚　辉　　陕西省西北妇女儿童医院　　　　　　　　主任医师
　　梁　宁　　广西壮族自治区人民医院　　　　　　　　主任医师
　　崔满华　　吉林大学第二医院　　　　　　　　　　　主任医师
　　路军丽　　首都医科大学附属北京朝阳医院　　　　　主任医师

| 本篇引用的法规 |  |
| --- | --- |

| 中央库 |  |
| --- | --- |

[国家卫生健康委员会、国家发展改革委、教育部等关于印发加强和完善麻醉医疗服务意见的通知](https://www.pkulaw.com/chl/78ca6b86c27f73a7bdfb.html)

© 北大法宝：（[www.pkulaw.com](https://www.pkulaw.com/)) 专业提供法律信息、法学知识和法律软件领域各类解决方案。北大法宝为您提供丰富的参考资料，正式引用法规条文时请与标准文本核对。
欢迎查看所有[产品和服务](https://www.pkulaw.com/helps/2.html)。法宝快讯：[如何快速找到您需要的检索结果？](https://www.pkulaw.com/helps/70.html)[法宝](https://www.pkulaw.com/helps/69.html)V6有何新特色？

扫描二维码阅读原文

**原文链接：**<https://www.pkulaw.com/chl/962bf417641e5f6dbdfb.html>
